# Cerebral venous thrombosis: imaging patterns

**DOI:** 10.1590/0100-3984.2021.0019

**Published:** 2022

**Authors:** Isabela Magalhães Oliveira, Juliana Ávila Duarte, Mariana Dalaqua, Vinicius Menezes Jarry, Fernanda Veloso Pereira, Fabiano Reis

**Affiliations:** Department of Radiology, Universidade Estadual de Campinas (Unicamp), Campinas, SP, Brazil.; Department of Radiology and Diagnostic Imaging, Hospital de Clínicas de Porto Alegre (HCPA), Porto Alegre, RS, Brazil.; Department of Diagnostic and Interventional Imaging, Hôpital du Valais, Sion, Valais, Switzerland.

**Keywords:** Intracranial venous thrombosis/diagnostic imaging, Tomography, X-ray computed, Magnetic resonance imaging, Trombose venosa intracraniana/diagnóstico por imagem, Tomografia computadorizada, Ressonância magnética

## Abstract

Cerebral venous thrombosis (CVT) is an uncommon condition that is potentially reversible if properly diagnosed and promptly treated. Although CVT can occur at any age, it most commonly affects neonates and young adults. Clinical diagnosis is difficult because the clinical manifestations of CVT are nonspecific, including headache, seizures, decreased level of consciousness, and focal neurologic deficits. Therefore, imaging is crucial for the diagnosis. Radiologists should be able to identify the findings of CVT and to recognize potential imaging pitfalls that may lead to misdiagnosis. Thus, the appropriate treatment (anticoagulation therapy) can be started early, thereby avoiding complications and unfavorable outcomes.

## Abstract (pt)

A trombose venosa cerebral (TVC) é uma condição incomum que é potencialmente reversível se diagnosticada corretamente e prontamente tratada. Embora a TVC possa ocorrer em qualquer idade, ela afeta mais comumente neonatos e adultos jovens. O diagnóstico clínico é difícil porque as manifestações clínicas da TVC são inespecíficas, como cefaleia, convulsões, diminuição do nível de consciência e déficits neurológicos focais. Nesse contexto, a imagem é crucial para o diagnóstico e os radiologistas devem ser capazes de identificar os achados de TVC e reconhecer potenciais armadilhas de imagem que podem levar a diagnósticos incorretos. Portanto, o tratamento adequado (terapia anticoagulante) deve ser iniciado precocemente para evitar complicações e desfechos desfavoráveis.

## INTRODUCTION

Cerebral venous thrombosis (CVT) is defined as the presence of a thrombus within a venous sinus, superficial intracranial vein, or deep intracranial vein. It is an uncommon condition that is potentially reversible if diagnosed and treated appropriately and promptly. It typically affects young females, correlating with common prothrombotic states (such as Behçet’s disease), the use of oral contraceptives, adjacent infections, trauma, intracranial hypotension, and the peripartum period. Because patients with CVT can present with a wide spectrum of nonspecific manifestations, including headache, focal neurologic deficits, seizures, and decreased level of consciousness, the condition is difficult to diagnose clinically. Consequently, imaging is fundamental to its diagnosis. Therefore, radiologists should be able to recognize this condition, allowing appropriate treatment (anticoagulation therapy), which can reverse the disease process as well as significantly reducing the risk of acute complications and long-term sequelae(^1^,^2^).

**Figure 1:**
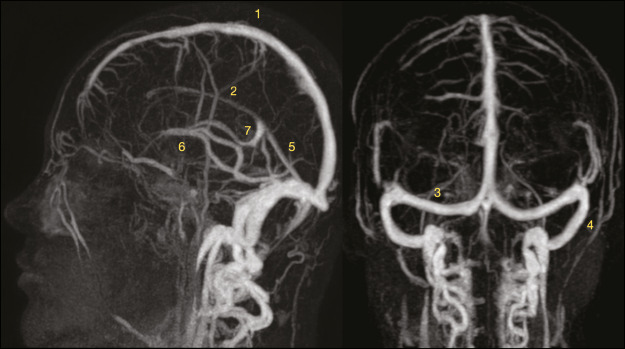
Intracranial contrast-enhanced MR venogram showing the anatomy of the cerebral venous system, consisting of the deep venous system, superficial venous system, and dural venous sinuses (with superior and inferior components). The superior dural sinuses comprise the superior sagittal sinus (1), the inferior sagittal sinus (2) and the straight sinus (5)—which converge at the torcular herophili—as well as the transverse sinus (3) and sigmoid sinus (4). The inferior dural sinuses comprise the cavernous sinuses, which are connected through the anterior and posterior intercavernous sinuses, and the superior and inferior petrosal sinuses, which drain toward the sigmoid sinus and jugular bulb. The deep system includes the vein of Galen (7), the internal cerebral veins (6), and their tributaries; the Rosenthal veins (basal veins) and its tributaries; and the medullary and subependymal veins, which drain the hemispheric white matter.

The goals of this article are to illustrate the anatomy of the cerebral venous system (Figure 1); describe the various CVT subtypes; review the main imaging findings of CVT and associated parenchymal abnormalities; highlight the predisposing factors for CVT; and demonstrate the diagnostic pitfalls. The cases presented are from the radiology departments of the participating institutions.

## PREDISPOSING FACTORS

There is a wide range of causes and risk factors associated with CVT. Causal factors may be local (related to intrinsic or mechanical conditions of the venous system) or systemic (related to prothrombotic states). Local processes that alter venous flow include sinus trauma, regional infection, and neoplastic invasion or compression. Systemic causes include protein S and protein C deficiencies, as well as a peripartum state, oral contraceptive use, and malignancy. In as many as 25% of cases, no cause is identified^(3)^.

## CLINICAL MANIFESTATIONS

The clinical manifestations of CVT vary depending on the extent, location, and time since the onset of the venous thrombotic process, as well as on the venous collateral circulation. They fall broadly into three categories: signs and symptoms of raised intracranial pressure; a focal brain lesion; and a combination of the two. Headache is the most common symptom, although seizures and focal neurological manifestations may also be observed. The time from the onset of the process to the appearance of symptoms varies widely^(4)^: 2–30 days (subacute presentation) is the most common range, followed by < 2 days (acute presentation) and > 30 days (chronic presentation).

## BEST IMAGING MODALITY

It is well established that the current gold standard to depict CVT is the combination of conventional magnetic resonance imaging (MRI) with some kind of magnetic resonance venography, particularly with dynamic time-resolved angiographic techniques, such as time-resolved imaging of contrast kinetics (TRICKS) and time-resolved imaging with stochastic trajectories. The TRICKS modality uses extremely rapid acquisitions to provide dynamic images of intravascular contrast flow. The use of TRICKS provides excellent spatial resolution, as well as dynamic flow information that has not been previously obtained without more invasive studies, such as interventional angiography^(5)^. The combination of conventional MRI and TRICKS demonstrates the location and extent of the thrombus, as well as the patent venous drainage pathways, the temporal evolution of the thrombosis, and associated brain abnormalities^(6)^.

## IMAGING FINDINGS

The imaging findings of CVT are related not only to the actual intraluminal thrombosis but also to the consequences of the obstructed venous drainage, such as parenchymal disturbances due to venous congestion and parenchymal or subarachnoid hemorrhage, as well as late-phase complications.

### Unenhanced CT

**Figure 2:**
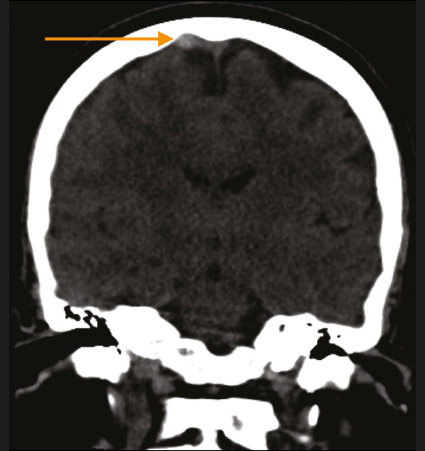
Coronal reconstruction of an unenhanced CT image showing hyperattenuation in the occluded cortical vein—the so-called “cord sign” (arrow).

Direct signs of CVT are seen in only one third of cases and are rarely visible on unenhanced CT scans. The “cord sign” (Figure 2) represents direct visualization of an acute hyperdense thrombus. More often, unenhanced CT shows only indirect signs of CVT, which may include diffuse brain edema and venous infarction(^1^,^6^).

### Contrast-enhanced CT

Direct evidence of CVT on contrast-enhanced CT includes the “empty delta sign”, which consists of a triangular area of contrast enhancement in the dural sinus surrounding the hypodense thrombus.

**CT venography**

In cases of CVT, direct visualization of the thrombus as a flling defect can be achieved with CT venography.

## MRI

**Figure 3:**
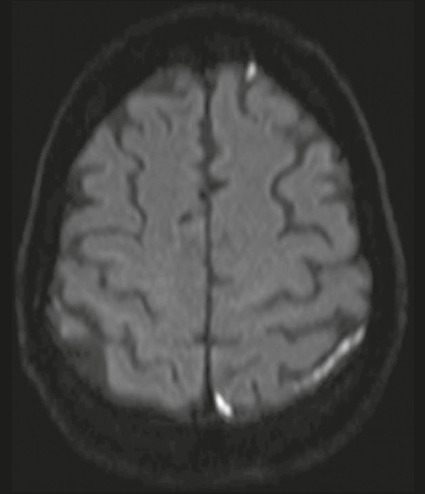
Axial diffusion-weighted image showing hyperintensities in cortical veins. An apparent diffusion coefficient map (not shown) confirmed true restricted diffusion.

On conventional MRI sequences, patent dural sinuses often appear as a flow void, due to the normal fast flow, whereas CVT may manifest as the absence of a flow void, which is often best seen on T2/fluid-attenuated inversion recovery (FLAIR) images. However, it should be emphasized that this finding is not specific for thrombosis and may be associated with transient hemodynamic conditions of slow flow, without thrombosis. On T1-weighted imaging (T1WI), a thrombus with methemoglobin is hyperintense in the subacute phase. The use of susceptibility-weighted imaging improves the detection of acute-phase CVT because magnetic susceptibility of deoxyhemoglobin makes dural sinus CVT hypointense(^1^,^6^). On diffusion-weighted imaging, an increased signal corresponding to the presence of intravascular clots may be depicted, even in the absence of parenchymal lesions. The corresponding low apparent diffusion coefficient suggests restriction of the movement of water molecules (i.e., restricted diffusion) at the site of occlusion (Figure 3).

### MR venography

The modality known as MR venography may be performed with or without the use of a contrast agent. Time-resolved contrast-enhanced MR venography has the highest accuracy and takes advantage of luminal flling by contrast material rather than relying on the MR flow phenomena using the time-of-fight technique.

### Parenchymal abnormalities

**Figure 4:**
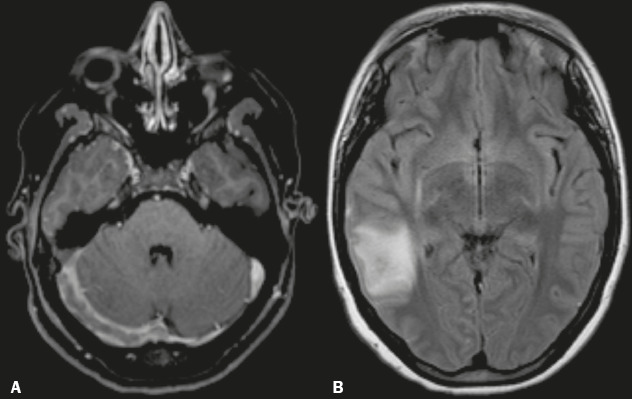
A 23-year-old woman with refractory headache. **A:** Axial MR angiogram showing a lack of flow in the enlarged right transverse sinus. **B:** Axial FLAIR MRI scan showing hyperintensity in the posterior right temporal lobe, consistent with vasogenic edema of a venous infarct.

**Figure 5:**
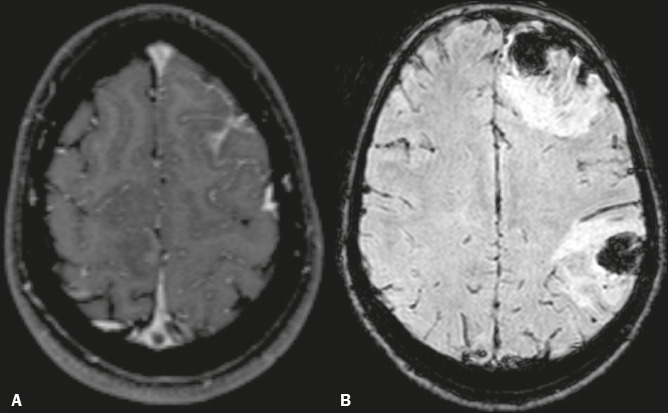
A 15-year-old female patient with CVT. A: Axial contrastenhanced three-dimensional T1-weighted magnetization prepared rapid gradient echo MRI sequence showing a central triangular flling defect (“empty delta sign”) in the superior sagittal sinus. B: Axial susceptibility-weighted MRI showing intraparenchymal hemorrhages in the frontal and parietal lobes.

**Figure 6:**
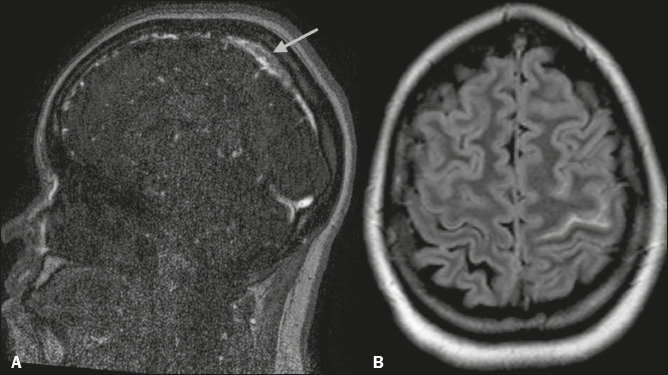
A 20-year-old woman with right-sided headache and hypoesthesia. A: Sagittal MR angiogram showing a lack of flow in the superior sagittal sinus (arrow). B: Axial FLAIR MRI scan showing subarachnoid hemorrhage in the left central sulcus.

In CVT, vasogenic and cytotoxic edema may be observed and are occasionally seen together. Like intraparenchymal hemorrhage, they can be located in subcortical territories, rather than in the typical arterial vascular distribution. Vasogenic edema, which may be reversible, develops secondary to elevated retrograde venous pressure (Figure 4), whereas cytotoxic edema appears if the cerebral perfusion pressure decreases and provokes tissue distress. Hemorrhages in the frontal and parietal lobes are often associated with superior sagittal sinus CVT (Figure 5), whereas those in the temporal and occipital lobes are more characteristic of transverse sinus CVT. In rare cases, CVT can be accompanied by subarachnoid hemorrhage (Figure 6) in the subacute phase. The pathogenic mechanism is venous hypertension secondary to retrograde pressure due to blocked cerebral venous system, resulting in bleeding into the subarachnoid space^(7)^.

## SUBTYPES OF CVT

### Dural sinus thrombosis

In most patients with CVT, multiple dural venous sinuses are involved. Those most commonly affected are the superior sagittal sinus, transverse sinus, and sigmoid sinus^(1)^.

### Isolated cortical vein thrombosis

Isolated cortical vein thrombosis is a relatively rare entity. Typical parenchymal findings are areas of focal cortical edema or hemorrhage, which may be nonspecific. On MR venography, an asymmetric absence of flow signal or reduced enhancement can be seen in the thrombosed cortical veins^(3)^.

### Deep CVT

**Figure 7:**
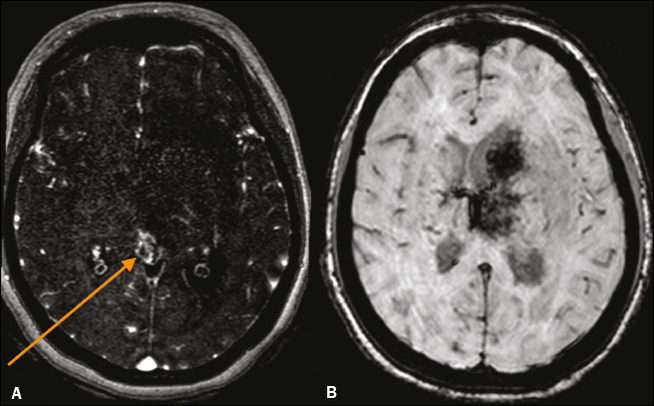
A 27-year-old woman with deep CVT. **A:** MR venogram showing a lack of flow in the internal cerebral veins (arrow), vein of Galen, and straight sinus (not demonstrated). **B:** Axial susceptibility-weighted MRI scan showing hemorrhagic transformation of a venous infarct in the left head of the caudate nucleus and thalamus.

Deep CVT is defined as thrombosis of the superior striate veins^(8)^, thalamostriate veins, internal cerebral veins, vein of Galen, or straight sinus. Thalamic involvement is the imaging hallmark of this condition, and it may involve the caudate nucleus and deep white matter (Figure 7)^(3)^.

### Chronic CVT

**Figure 8:**
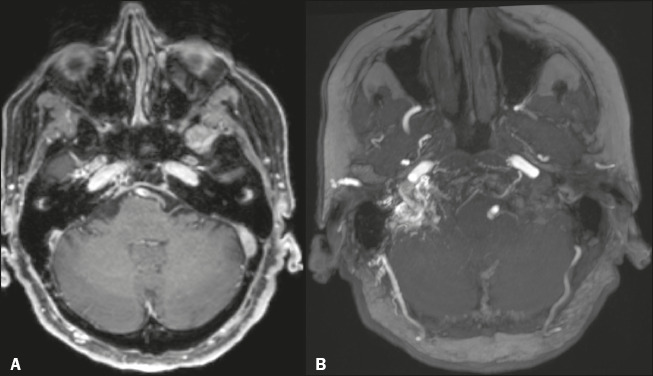
A 75-year-old female patient who underwent MRI for investigation of vertigo of undetermined origin. **A:** Axial contrast-enhanced three-dimensional (3D) T1-weighted magnetization prepared rapid gradient echo MRI sequence showing a flling defect in the transition between the right transverse and sigmoid venous sinuses. **B:** Axial maximum intensity projection reformatting of an unenhanced 3D time-of-fight MR angiogram, showing prominence of the right occipital and middle meningeal arteries, as well as branches of the right vertebral and ascending pharyngeal arteries (not shown) supplying a dural arteriovenous fistula consequent to chronic CVT with incomplete recanalization.

Chronic CVT with incomplete recanalization may present a diagnostic challenge on MRI. Although the thrombus typically has a signal that is isointense on T1WI and isointense/hyperintense on T2WI, there is significant variability in the signal intensity. Other findings that may be seen on MR venography are an irregular appearance of the sinus, with multiple serpiginous intrathrombus flow voids and dural collateral vessels, which are characteristics of incomplete recanalization^(4)^. One major complication of chronic venous thrombosis is dural arteriovenous fistula (Figure 8). On follow-up imaging, radiologists should look for dilated cortical arteries and veins in the surrounding affected parenchyma, which may be related to arterial recruiting and venous hypertension, respectively^(9)^.

## PREDISPOSING FACTORS FOR CVT

### Adjacent infections

**Figure 9:**
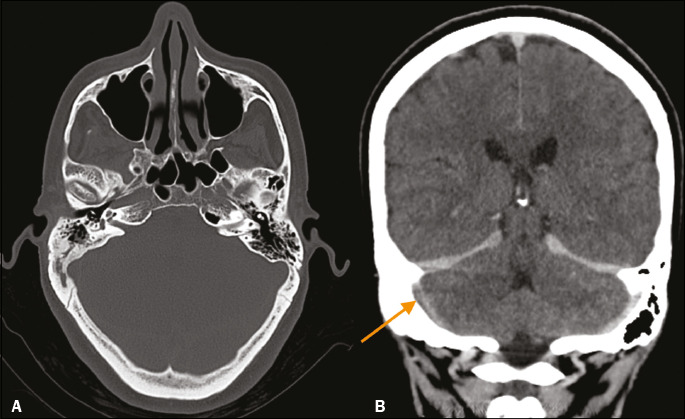
A 52-year-old woman complaining of holocranial headache. **A:** Axial CT scan showing opacification of some right mastoid air cells associated with bone sclerosis, indicative of chronic mastoiditis. **B:** Coronal CT angiogram showing a flling defect in the ipsilateral transverse sinus (arrow).

Otitis, mastoiditis, sinusitis, and meningitis can lead to CVT. Mastoiditis is the most common temporal bone infection and may cause septic thrombosis due to suppurative direct spread, sometimes requiring surgical intervention. Infection may also induce increased production of procoagulant compounds, resulting in a prothrombotic state and leading to dural venous sinus thrombosis (Figure 9).

### Prothrombotic states

**Figure 10:**
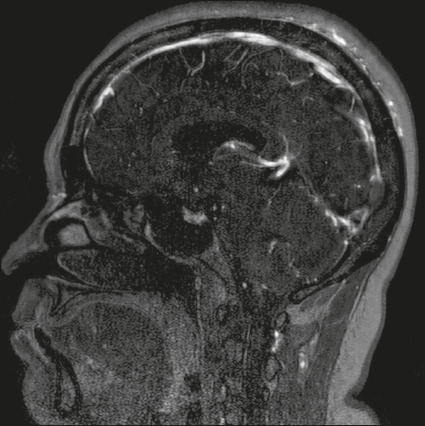
A 43-year-old woman with Behçet’s disease and persistent headache. Sagittal MR venogram showing a lack of flow in the torcular herophili.

Rheumatologic diseases such as systemic lupus erythematosus and Behçet’s disease may induce CVT. Behçet’s disease is a chronic, multisystem, relapsing infammatory perivasculitis. Although neurological involvement (neuro-Behçet’s disease) is a rare manifestation, it is one of the most serious causes of long-term morbidity and can result in cerebral venous thrombosis(^10^,^11^), as depicted in Figure 10.

### Prior trauma or surgery

**Figure 11:**
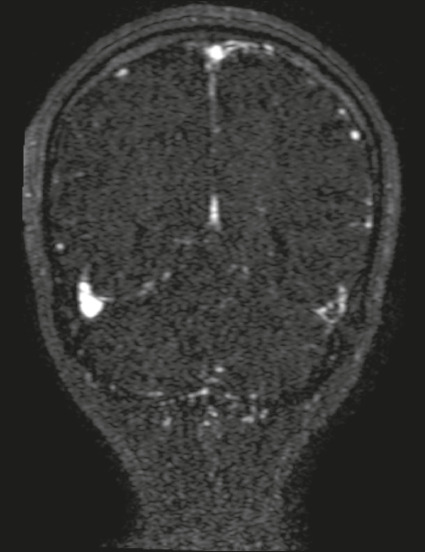
A 40-year-old man with a closed head injury and skull fractures who presented with a three-day history of a decreased level of consciousness. An unenhanced axial CT scan (not depicted) showed a hyperdense thrombus in the left transverse sinus. A coronal MR venogram (depicted) confirmed the corresponding lack of venous flow.

It is known that CVT is an underdiagnosed complication of trauma (Figure 11) and surgery. The prevalence of CVT is much higher in patients with skull fractures adjacent to a venous sinus, regardless of age group. Because the occurrence of traumatic CVT is usually not immediate, early imaging can miss that. In this context, contrast-enhanced follow-up CT or MR angiography is important to distinguish true thrombosis from sinus narrowing or occlusion caused by a compressing epidural hematoma^(12)^.

### Intracranial hypotension syndrome

**Figure 12:**
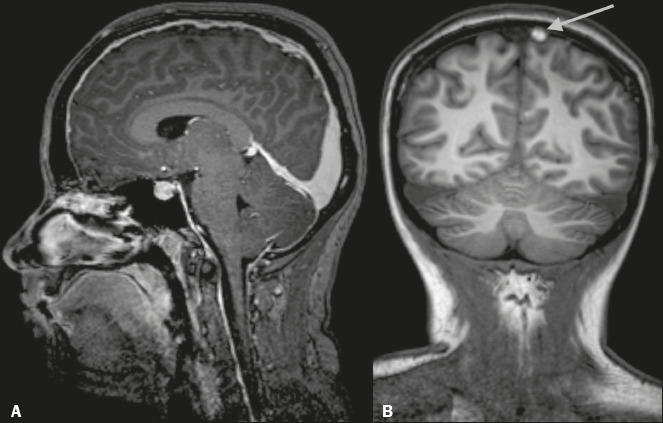
Cortical CVT in a 22-year-old woman who developed intracranial hypotension after epidural anesthesia. A: Sagittal contrast-enhanced three-dimensional T1WI showing dilated venous sinuses and enlargement of the pituitary gland (findings typical of intracranial hypotension). B: Unenhanced coronal T1WI showing a hyperintense thrombus in a cortical vein (arrow).

Intracranial hypotension syndrome consists of decreased cerebrospinal fluid volume, resulting in compensatory distention of vessels and downward traction of richly innervated structures. Dural venous sinus and cortical vein thrombosis have been described as uncommon complications of intracranial hypotension syndrome (Figure 12) and are believed to be secondary to slow blood flow in a dilated dural sinus. The risk of the syndrome is increased in patients with thrombotic preconditions(^13^,^14^).

## DIAGNOSTIC PITFALLS

There are pitfalls associated with all imaging techniques.

### Anatomical variations

Sinus asymmetry is mainly observed in the transverse sinuses. If the corresponding jugular foramen is also small, a narrow sinus may be considered a normal anatomical variation^(15)^.

### Arachnoid granulations

Arachnoid granulations are common projections of the subarachnoid space into a sinus, usually round and not occupying the entire sinus like CVT does.

### Increased hematocrit levels

In newborns with persistence of fetal hemoglobin and in situations such as dehydration and polycythemia, the sinuses and veins may appear relatively hyperdense on unenhanced CT, due to increased hematocrit levels. However, in such cases, the arteries will have a similar appearance^(15)^.

### Diffuse brain edema

Parenchymal hypodensity caused by diffuse brain edema may also make the venous structures appear relatively hyperdense on CT.

### Flow artifacts on MRI

Slow flow leads to inconsistent flow void^(15)^. Another flow pitfall is the “entry section phenomenon”, which is a flow-related enhancement artifact dependent on flow direction. Contrast-enhanced studies clarify the diagnosis.

## CONCLUSION

Albeit relatively uncommon, CVT is a potentially life-threatening disease. Imaging plays a crucial role in the diagnosis and identification of the patterns of CVT. Radiologists should be aware of the potential pitfalls in order to avoid misdiagnosis and allow early treatment, which is essential to decrease the rate of complications—such as dural arteriovenous fistula—and sequelae.
